# Tenofovir substitution in Namibia based on an analysis of the antiretroviral dispensing database

**DOI:** 10.1186/s40545-015-0034-6

**Published:** 2015-04-20

**Authors:** Francis Kalemeera, Assegid T Mengistu, Johannes Gaeseb

**Affiliations:** Pharmacology & Pharmacy Policy and Practice, School of Pharmacy, Faculty of Health Sciences, University of Namibia, 340 Mandume Ndemufayo, Windhoek, 9000 Namibia; Therapeutics Information and Pharmacovigilance Centre, National Medicines Regulatory Council, Ministry of Health and Social Services, Harvey Street, Windhoek, 9000 Namibia

**Keywords:** Tenofovir, Substitution, Adverse reactions, Namibia, Tubulopathy, Renal insufficiency

## Abstract

**Objectives:**

In the management of HIV infection, tenofovir is preferred to its predecessors – zidovudine and stavudine – in the antiretroviral therapy (ART) nucleoside backbone. Tenofovir’s (TDF) preference is based on its safety profile. Nevertheless, TDF causes adverse reactions, some of which warrant its substitution for patients. The rate of TDF-substitution is suggestive of the rate of occurrence of TDF-related adverse reactions. However, the rate of substitution of TDF with another nucleoside reverse transcriptase inhibitor (NRTI) in Namibia was unknown. The objective of this review was to measure the rate of TDF’s substitution for the period of January 1, 2008 to November 30, 2011, and to compare the gender difference in the rates of TDF’s substitution.

**Methods:**

We accessed antiretroviral medicine dispensing records from the national antiretroviral dispensing database (NDB). We selected patients who were started on a TDF-containing conventional ART regimen – 2NRTI+1NNRT. We used the initial and current ART regimens to identify records of TDF’s substitution with another NRTI.

**Results:**

A total of 84,741 patients were initiated on ART (Jan-1-2008 to Nov-30-2011). A total of 52,612 patient-records were excluded from the analysis because they did not meet the criteria for inclusion. Of the 32,129 included records, 59.4% (n=19 096) and 40.6% (n=13 033) were for female and male patients, respectively. Of these, 1.2% (n=380) of the patients had their TDF substituted with another NRTI. Of the females and males, respectively, 1.1% (95% CI: 0.9-1.3; n=210) and 1.3% (95% CI: 1.1-1.5; n=170) had TDF substituted with another NRTI. No gender difference was observed (p-value = 0.11).

**Conclusion:**

The percentage of patients for whom TDF was substituted with another NRTI, possibly due to TDF-related adverse reactions, was within the current published limits. However, 1.2% is likely not a true representation of the percentage of patients who experience adverse events because some patients could have been maintained on TDF even in the presence of adverse events. Further investigation is required to determine the clinical reasons for TDF’s withdrawal.

## Introduction

Tenofovir (TDF) is an acyclic nucleotide analogue that is used for the treatment of the human immunodeficiency virus (HIV) infection. TDF in combination with lamivudine (3TC) is effective against Hepatitis-B virus, making the TDF/3TC nucleoside backbone a better choice for Hepatitis-B/HIV co-infected patients [1]. TDF is a nucleotide reverse transcriptase inhibitor, but it is also classified as a nucleoside reverse transcriptase inhibitor (NRTI) [2]. In this paper TDF is referred to as an NRTI.

Currently, the combination of TDF and 3TC or emtricitabine (FTC) is the preferred nucleoside backbone of the first line antiretroviral therapy (ART) [3,4]. Previously, stavudine-(d4T) combined with 3TC was the preferred NRTI backbone of first-line ART. However, due to an increased incidence of d4T-associated toxicities, including: peripheral neuropathy, lipoatrophy, lactic acidosis, and pancreatitis [5-7], d4T was replaced by zidovudine (AZT). Similarly, AZT’s place was taken by TDF due to a relatively high incidence of AZT-associated anaemia, and less frequently lipoatrophy. Because the TDF-based first line ART regimen was as effective as the d4T- and AZT-based regimens, and also because the cost of acquisition of TDF was low [8], TDF’s position in the NRTI backbone of the first-line ART regimen was secured by TDF’s better safety profile. Nevertheless, TDF is known to cause adverse reactions in the gastrointestinal tract, renal system, and skeleton system. In the skeletal system, TDF has been shown to be associated with bone demineralisation, which is measured through the use of Dual Energy X-ray Absorptiometry (DEXA). Some adverse reactions may be more common in females than in males, as is the case for liver related reactions [9,10].

In the kidneys, TDF destabilises the re-absorptive capacity and secretory function of the proximal tubules resulting in the loss of filtered compounds such as glucose, proteins and phosphate in the urine, coupled with the reduced secretion of protons and TDF [11]. Recent evidence suggests that TDF-associated proximal tubulopathy is due to mitochondrial toxicity. This evidence is founded on biopsy results that have shown mitochondrial damage in the proximal tubular cells in patients with renal insufficiency while taking TDF-containing ART. It is believed that inhibition of mitochondrial DNA-polymerase gamma, an NRTI-class related mechanism of mitochondrial dysfunction, underlies TDF-associated proximal tubulophathy [1,11,12].

In 2010, TDF + 3TC became the preferred NRTI backbone for first line ART in Namibia. Previously, TDF had been preserved for second line ART. Eventually, a large number of patients were started on TDF-based ART. Like its predecessor antiretroviral medicines, TDF is associated with serious adverse reactions that call for its withdrawal should they occur. In this regard, the frequency of substitution of TDF with another NRTI is suggestive of a TDF-associated adverse reaction. Another possible reason for substitution of TDF with another NRTI would possibly be treatment failure. However, the rate of TDF’s withdrawal and substitution, in Namibia, was unknown. Consequently, we reviewed the National ART dispensing database of Namibia to provide insight into the percentage of patients who may not tolerate TDF.

## Objectives

The primary objective of this review was to assess the rates of substitution of TDF with another NRTI as first line ART, and the secondary objective was to compare the gender difference in the rates of substitution of TDF with another NRTI.

## Methods

### Study design

In this retrospective cohort study, we assessed antiretroviral dispensing records of patients who were started on ART from January 1, 2008 to November 30, 2011. The National Antiretroviral Database (NDB) was the sole source of records. The primary outcome was the substitution of TDF with another antiretroviral medicine, while the Co-administered medicines remained unchanged.

### Inclusion and exclusion criteria

All public health facilities that provided ART in Namibia from January 2008 to November 31, 2011 were included. The ART dispensing records of interest were for patients who were initiated on the conventional first line regimen, i.e., 2NRTI + 1NNRTI, in the period stated. The two regimens were TDF/3TC combined with EFV or NVP. Records for adult patients, as defined by the World Health Organisation (WHO), were included. Records that indicated the withdrawal of TDF and the NNRTI were excluded, because that type of substitution indicated the occurrence of treatment failure. Also, we excluded records of patients who were on TDF-sparing regimens at the start of ART; and records of those with non-conventional starting regimens even though they were TDF-containing, because some of them were second-line regimens and clinical justification for their use was not available.

### Study procedures

We retrieved the automated records of the dispensing of antiretroviral medicines from the national database in an Excel format for the period of January 1, 2008 to November 30, 2011. In regards to antiretroviral medicines dispensed, the national database provided the starting and current ART regimens. The records which were included in the analysis were for patients who were started on TDF/3TC/NVP or EFV. We identified records of those for whom TDF was replaced by another NRTI.

### Ethical approval

Patient consent was not sought because patient information was sourced from existing databases and clinical records without requiring additional information directly from patients. ART dispensing data were captured in the national database housed in the Ministry of Health and Social Services’ Division of Pharmaceutical Services. Only TIPC staff members mandated by the Ministry of Health and Social Services to implement medicines safety analyses were involved in the medical record abstraction process and in the epidemiological study considered to be a public health activity. Precautionary measures were followed to maximize the confidentiality by removing all personal identifying information.

### Analysis

We used descriptive methods, and the Students-T test for the comparison between female and male patients’ TDF-substitution rates. We set the confidence level at 95% and the statistical significance at a p-value of <0.05.

## Results

A total of 84,741 patients were started on ART from January 1, 2008 to November 30, 2011. Of these 50,252 patients was started on TDF-sparing regimens, and so were excluded from the analysis. Furthermore, we excluded 326 patients younger than 18 years of age at the start of ART, leaving 34,163 records. Amongst these, 2,034 patients were started on either a non-conventional first line ART regimen or on second line therapy, and so they were excluded from the analysis, leaving a total of 32,129 patient records for analysis. Of these 32,129 patients 59.4% (n = 19,096) were female and 40.6% (n = 13,033) were male.

### Distribution according to year of ART initiation

Of the 32,129 patients who were started on TDF/3TC/EFV or TDF/3TC/NVP the number of persons by year is as follows: 2,865 in 2008; 3,497 in 2009; 8,666 in 2010; and 17,101 in 2011 (Table 1).

**Table 1:** **Comparison between female and male percentages of patients changed from TDF to another NRTI**

**Period (Years)**	**Number (percentage) of patients started on ART**			**Comparison between females and males: Number (Percentage: confidence interval of patients changed from TDF**		
	**Total (N)**	**Female: n (%)**	**Male: n (%)**	**Female**	**Male**	**p-value**
**2008**	2,898	1,642 (56.7)	1,256 (43.3)	18 (1.1: 0.6 – 1.6)	5 (0.4: -0.1 – 0.9)	0.03
**2009**	3,536	1,889 (53.4)	1,647 (46.6)	32 (1.7: 1.1 – 2.3)	18 (1.1: 0.5 – 1.7)	0.13
**2010**	8,666	5,100 (58.9)	3,566 (41.1)	71 (1.4: 0.9 – 1.9)	70 (1.9: 1.7 – 2.6)	0.01
**2011**	17,101	10,465 (61.2)	6,636 (38.8)	89 (0.8: 0.5 – 1.1)	77 (1.2: 0.9 – 1.5)	0.01
**TOTAL**	**32,129**	**19,096 (59.4)**	**13,033 (40.6)**	**210 (1.1: 0.9 – 1.3)**	**170 (1.3: 1.1 – 1.5)**	**0.11**

### Number and percentage of patients for whom TDF was substituted

**Figure 1:**
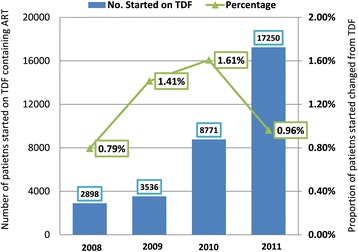
Percentage of patients changed from TDF to another NRTI.

Of the 32,129 patients, 1.2% (n = 380) patients had TDF substituted with another NRTI leaving 31,749 on TDF-based ART (Table 1). Of those for whom TDF was substituted, 210 and 170 were female and male, respectively (Table 1). The percentage (and number) of substitutions of TDF with another NRTI that were implemented per year, from 2008 to 2011, was as follows: 0.80% (n = 23) in 2008; 1.43% (n = 50) in 2009; 1.63% (n = 141) in 2010; and 0.97% (n = 166) in 2011. (Figure 1, Table 1). The overall percentage of female patients that were changed from TDF to another NRTI was 1.1% [CI 0.9-1.3] and for the males it was 1.3% [CI 1.1-1.5], (p-value = 0.11).

## Discussion

In settings with relatively efficient availability and accessibility of antiretroviral medicines, such as Namibia [4], the most likely reason for substitution of TDF with another NRTI is occurrence of severe adverse reactions. While the reasons for substitution were not assessed in this study, it is likely that the replacement of TDF with another NRTI without alteration to the other antiretroviral medicines in the regimen was prompted by TDF-associated adverse reactions. The percentage of patients, for whom TDF was substituted with another NRTI from 2008 to 2011 was low (1.2%: 380 out of 32 129). This value is somewhat lower than in published literature: 5.5% for all causes [13]; 3% for renal events [14]; and 28% for bone demineralisation [9]. According to the spontaneously reported adverse event data in Namibia’s national pharmacovigilance centre, TDF was reported to be associated with intractable diarrhoea, intractable vomiting, and renal impairment, but not osteopenia, osteoporosis, or fractures (unpublished observations).

Since the Namibia ART guidelines that were in effect from 2008 to 2011 recommended the substitution of TDF with another NRTI in the event of occurrence of a severe adverse reaction [15,16], it is plausible that substitution of TDF with another NRTI was prompted by adverse reactions. According to TDF’s prescribing information, diarrhoea, nausea, vomiting and dyspepsia are listed amongst TDF-associated gastrointestinal (GI) side effects [1]. The presentation of GI side effects is usually clear, and they can guide the decision to continue or stop administration of TDF. For example: mild to moderate gastrointestinal effects may not, but more serious ones such as intractable vomiting and intractable diarrhoea, may necessitate TDF’s substitution with another NRTI. In all cases, the decision to withdraw TDF should be dependent on the appropriate assessment of causality of the event, including the ruling-out of possible non-drug causes. TDF-associated GI side effects were amongst the reasons for TDF’s withdrawal, which is evidenced by reports in TIPC’s database of adverse reactions (unpublished observation).

In some instances, TDF-associated renal insufficiency is slow and conspicuous in development such that its early detection necessitates frequent renal function tests (RFT) [17,18]. Since the Namibia’s ART guidelines recommended regular RFTs – particularly, the calculation of the GFR – for patients on TDF-containing ART, we believe that the detection of renal impairment was one of the major reasons for TDF’s substitution [18]. The manifestation of reduced GFR was not considered a pre-requisite for substitution of TDF according to Namibia’s ART guidelines. In such instances, TDF’s dosing interval would be lengthened, thus fostering the pathological process of renal tubular damage, if indeed TDF was the primary cause of renal insufficiency [18]. On this basis, there is reason to believe that the substitution of TDF, due to abnormal RFT was prompted by a very low GFR <30mls/minute. At times, patients taking TDF-based ART experience acute renal failure and the Fanconi Syndrome [1,19], and TDF’s prompt and permanent withdrawal would be implemented [13]. Evidence of TDF-associated renal insufficiency as a cause for TDF’s withdrawal is available in TIPC’s database of adverse reactions (unpublished observation). In regards to gender, our results suggest that gender did not have any influence on the rate of substitution from TDF to another NRTI (p-value = 0.11).

TDF is known to reduce bone mineral density resulting in osteopenia and osteoporosis, which could be secondary to TDF-associated renal impairment [1,20]. The pain associated with bone fragility and the resultant fractures are a strong basis for TDF-substitution. While a decrease of bone mineral density as a TDF-related adverse effect is mentioned in Namibia’s ART guidelines, the use of DEXA to measure bone demineralisation in patients taking TDF is not recommended in Namibia’s ART guidelines. This is possibly due to the fact that DEXA is only available in private practice settings in Namibia. Therefore, it is not possible to assume that osteopenia, osteoporosis, and the resultant fractures contributed to the substitution of TDF.

It is unlikely that treatment failure was a reason for TDF’s withdrawal and replacement with another NRTI. This is because Namibia’s ART guidelines that were in force at the time of this review recommended that TDF and 3TC should be maintained in second line therapy, while EFV or NVP are replaced with LPV-r and AZT [18]. Moreover, for the 380 patients whose TDF was substituted with another NRTI, EFV- or NVP were retained on the ART regimen thus ruling out the plausibility that treatment failure had occurred.

Our findings were limited by a number of issues. First: we eliminated from the analysis patients who were changed from a TDF-containing ART regimen to a non-conventional TDF-sparing ART first line regimen, yet amongst these some may have experienced a TDF-related adverse reaction. Secondly: because renal insufficiency is not a pre-requisite to substitute TDF until the GFR is <30mls/min, it is possible that a number of patients, who were still on TDF at the time of this review, may have developed renal insufficiency, but still at a low grade such that TDF was maintained. Moreover, the NDB lacked data on the adjusted dose of TDF so that patients with reduced GFR could not be identified. Lastly: our analysis was based on data from the NDB, which lacked information on the adverse reactions that the patient may have experienced. Therefore, it was not possible to allocate percentages of patients to GI-, renal-, and bone- related adverse events.

## Conclusion

The percentage of patients for whom TDF was replaced with another NRTI, possibly due to adverse reactions, was within the current published values. There was no significant difference between TDF substitution between females and males. Since the ART guidelines recommended that following the detection of renal insufficiency of lower severity grades, TDF’s dose was to be recalculated and its administration maintained, it is possible that some patients with renal insufficiency remained on TDF. Furthermore, the paucity of tests to assess bone mineral density means that patients who may have had osteopenia and osteoporosis associated with TDF could have remained on TDF-based therapy. Therefore, the estimated percentage (1.2%) of patients whose TDF was replaced with another NRTI is unlikely to be a true representation of the percentage of patients that do not tolerate TDF in Namibia. Further analysis is required to determine the actual events that led to TDF’s withdrawal, and to review patient treatment notes for complaints of bone pain and events of fractures. Also, there is need to review data for patients who were initiated on TDF-based first line therapy when they were younger than 18 years of age. Further studies are needed to estimate the prevalence of different types of TDF associated adverse reactions; and to detect the average time of manifestation of these adverse events after initiation of ART.

## Abbreviations

3TC: Lamivudine
ART: Antiretroviral therapy
AZT: Zidovudine
D4T: Stavudine
EFV: Efavirenz
FTC: Emtricitabine
GFR: Glomerular filtration rate
HAART: Highly active antiretroviral therapy
HIV: Human immunodefficiency virus
IDV-r: Indinavir-ritonavir
LPV-r: Lopinavir-ritonavir
NDB: National antiretroviral dispensing database
NRTI: Nucleoside reverse transcriptase inhibitor
NNRTI: Non-nucleoside reverse transcriptase inhibitor
NVP: Nevirapine
*TDF*: *Tenofovir*
